# Supplementary material for: Clinical Burden and Healthcare Utilization Associated with Hospitalizations of RSV-Infected Polish Children During the 2022/23 Season
Source: Viruses. 2025 Dec 30;18(1):60. doi: 10.3390/v18010060 (PMC12846529; doi:10.3390/v18010060)
Supplement: Supplementary file 1 [file viruses-18-00060-s001.zip › viruses-3993611-supplementary.pdf]

Supplementary Table 1. Hospitals and patients included in the study.

| Contract size* | Contract value* (%) | Overall number of hospitals | Included hospitals | | | Patient questionnaire A | | | Patient questionnaire B | | |
|---|---|---|---|---|---|---|---|---|---|---|---|
| | | | Period I | Period II | Overall | Period I | Period II | Overall | Period I | Period II | Overall |
| **High** | 38% | 49 | 9 | 6 | 15 | 246 | 169 | 415 | 84 | 68 | 152 |
| **Medium** | 51% | 190 | 10 | 16 | 26 | 275 | 362 | 637 | 133 | 134 | 267 |
| **Low** | 11% | 86 | - | - | - | - | - | - | - | - | |
| **Overall** | 100 % | 325 | 19 | 22 | 41 | 521 | 531 | 1052 | 217 | 202 | 419 |

* - contract between the hospital and the NHF for providing pediatric healthcare services

Supplementary Table 2. Diagnosis and treatment details

| **Health state at admission (mean)** | **All patients (N=419)** | **Patients aged ≤12 months (N=297)** | **Patients aged >12 months (N=122)** |
|---|---|---|---|
| Body temperature (°C) | 37.2 | 37.0 | 37.5 |
| $O_2$ saturation | 95 | 95.4 | 95.4 |
| **Additional diagnosis during RSV infection; weighted % (n)** | **All patients (N=419)** | **Patients aged ≤12 months (N=297)** | **Patients aged >12 months (N=122)** |
| RSV pneumonia | 56.8% (225) | 53.8% (157) | 60.8% (68) |
| Bronchiolitis associated with RSV | 35.9% (159) | 40.6% (123) | 29.5% (36) |
| RSV bronchitis | 12.4% (52) | 9.8% (30) | 15.8% (22) |
| Otitis media due to RSV | 3.8% (11) | 1.4% (4) | 7.0% (7) |
| Bacterial superinfection during RSV other than the above-mentioned | 2.3% (8) | 0.7% (2) | 4.4% (6) |
| Subglottic laryngitis in RSV | 0% | 0% | 0% |
| None of the above | 2.1% (8) | 1.4% (4) | 2.9% (4) |
| | | | |
| **Use of respiratory support during hospitalization; weighted % (n)** | **N=419** | **N=297** | **N=122** |
| Yes | 21.1% (103) | 24.6% (75) | 16.3% (28) |
| No | 78.9% (316) | 75.4% (222) | 83.7% (94) |

Supplementary Table 3. Pharmacotherapy applied during the hospital stay and after discharge; weighted % (n)

| Medications prescribed | During the hospital stay (n=417)* | After discharge (n=359)* |
|---|---|---|
| Inhaled steroid therapy | 67.6% (274) | 54.6% (193) |
| Antibiotic therapy | 61.5% (245) | 26.7% (78) |
| Inhalation of Beta2-mimetics + anticholinergics | 48.1% (191) | 22.8% (74) |
| Systemic steroid therapy | 44.3% (190) | 0.9% (3) |
| Inhalation of beta2-mimetics | 36.9% (155) | 12.0% (46) |
| Antipyretics/analgesics/NSAIDs | 33.2% (121) | 1.4% (5) |
| Inhalation of saline | 28.7% (124) | 26.5% (106) |
| Inhalation of hypertonic saline | 15.6% (68) | 13.9% (44) |
| Anticholinergic drugs | 13.0% (58) | 6.7% (25) |
| Mucolytics | 12.5% (41) | 10.5% (26) |
| Probiotics | 12.5% (45) | 7.8% (27) |
| Drops, nasal sprays | 8.2% (37) | 3.7% (9) |
| Antihistamines | 5.4% (17) | 2.6% (8) |
| Gastrointestinal drugs (nausea, vomiting, diarrhea, heartburn) | 4.8% (20) | 0% |
| Electrolytes/glucose | 4.0% (18) | 0% |
| Antifungal | 3.7% (18) | 1.2% (5) |
| Cough suppressants | 2.8% (8) | 0.6% (2) |
| Antiviral drugs | 0.9% (3) | 0% |
| Pharmacological treatment other than the above-mentioned | 4.5% (19) | 1.8% (8) |

* - patients who have been prescribed pharmacotherapy
